# Identification of Important Physiological Traits and Moderators That Are Associated with Improved Salt Tolerance in CBL and CIPK Overexpressors through a Meta-Analysis

**DOI:** 10.3389/fpls.2017.00856

**Published:** 2017-05-29

**Authors:** Yuanchun Ma, Qunkang Cheng, Zongming Cheng, Hui Li, Youhong Chang, Jing Lin

**Affiliations:** ^1^Jiangsu Key Laboratory for Horticultural Crop Genetic Improvement, Jiangsu Academy of Agricultural SciencesNanjing, China; ^2^Department of Plant Sciences, University of Tennessee, Knoxville, KnoxvilleTN, United States; ^3^Department of Entomology and Plant Pathology, University of Tennessee, Knoxville, KnoxvilleTN, United States

**Keywords:** *CBL* gene family, *CIPK* gene family, over-expression, salt tolerance, transgenic, meta-analysis

## Abstract

The CBL-CIPK pathway is a plant-specific Ca^2+^ sensor relaying pathway that has been shown to be involved in plant response to salt stress. Over-expression of CBL-CIPK network genes has been reported to increase salt tolerance in many studies. The studies on the overexpression of CBL-CIPK network genes, however, have used various indices to evaluate the effect of these genes on salt tolerance and have indicated a variety of roles for the major CBL-CIPK pathway genes. Therefore, it is of great interest to analyze the various effects resulting from the overexpression CBL-CIPK pathway genes and their relation to salt tolerance. The meta-analysis conducted in the present study investigated how over-expression of *CBLs or CIPKs* in transgenic plants affects the response to salt stress and identified the increase or decrease that occurs in these experimental variables when foreign *CIPK* or *CBL* genes are overexpressed in transgenic plants. The data from the collective studies on over-expression of *CIPK*s indicated that 6 of the 11 examined parameters (main effects) increased by 22% or more, while two of the six examined parameters increased by at least 78% in transgenic plants overexpressing *CBL* genes. In addition to analyzing the impact of overexpression on the main effects, eight different modifying parameters were also analyzed. Results indicated that several moderators impact the extent to which overexpression of *CBLs* and *CIPKs* affect the main parameters. The majority of *CBLs* have been obtained from dicotyledonous plants and most of the *CBLs* and *CIPKs* have been expressed in dicotyledonous plants. In comparison to homologous expression, the meta-analysis indicated that heterogeneous expression of *CBLs* resulted in greater increases in seed germination. The results of the meta-analysis provide information that could be useful in designing research to examine the mechanisms by which *CBL-CIPK* pathway genes increase salt tolerance in plants.

## Introduction

Salinity is one of the major abiotic stresses that limits the production and yields of agricultural crops worldwide (Cramer et al., 2011). Salt stress impacts plants at the molecular, physiological, and cellular levels during their life cycle (Ruiz-Lozano et al., 2012). Elucidating the plant parameters that respond to salt stress, and understanding the mechanisms involved in salt tolerance, can improve efforts to genetically engineer crops with improved levels of salt tolerance.

Many studies have examined the functional role of the CBL-CIPK pathway in plants in relation to plant response to abiotic stress (Xiang et al., 2007; Chaves-Sanjuan et al., 2014; Zhang et al., 2014; Pandey et al., 2015; Wang et al., 2016). The CBL-CIPK pathway is a Ca^2+^ sensor relaying pathway that has been demonstrated, along with the SOS signaling pathway, to be involved in plant response to salt stress. External stresses induce an increase in the concentration of intracellular Ca^2+^, and a CBL, a plant-specific sensor relay (Liu and Zhu, 1998; Kudla et al., 1999), senses the change in the Ca^2+^ level. CBL, accompanied by a CIPK then activates SOS1 by phosphorylation. *SOS1*, which belongs to the CPA1 (cation/proton antiporter 1) family of genes acts as a Na^+^/H^+^ antiporter to compartmentalize or exclude Na^+^ and maintain the balance of K^+^/Na^+^ (Zhu, 2002).

In addition to the SOS pathway, which is the classical pathway in the CBL-CIPK signaling network, also interacts with other signaling pathways to help plants cope with salt stress. Various members of the *CBL* and *CIPK* gene families are involved in salt tolerance. In *Arabidopsis*, CBL1, CBL9, CBL4, and CIPK6 regulate AKT-type K channels such as AKT1 or AKT2 (Li et al., 2006; Lee et al., 2007; Held et al., 2011; Nieves-Cordones et al., 2012). Studies have also reported that, in response to salinity stress, the CBL-CIPK system interacts with the ABA signaling pathway via the ABI2 protein (Jia et al., 2002; Ohta et al., 2003).

In response to salinity stress, plants maintain the ionic balance by excluding Na^+^ from cells or sequestrating Na^+^ through compartmentalization. Many studies have reported that overexpression of *CBL* or *CIPK* genes can increase salt tolerance and numerous members of these gene families have been overexpressed in different plant species (39 papers listed in **Appendix S1**). It has been difficult to distinguish, however, the precise mechanisms and different physiological processes that have been activated or suppressed by the overexpression of *CBLs or CIPKs* in transgenic plants subjected to non-stress or salt stress conditions because so many parameters are affected.

Meta-analysis represents a statistical synthesis of the results obtained in a collection of studies that have been systematically collected and appraised. The conformance and magnitude of a treatment effect is estimated much more accurately in a meta-analysis than it is from any single study alone. A meta-analysis is also able to describe the range of the impact of an effect and test which factors affect the magnitude of the effect size if the treatment effect is inconsistent in different studies. In the present study, a meta-analysis was conducted on the over-expression of CIPK-CBL pathway genes in order to characterize the effect of overexpression on the response of transgenic plants subjected to salt stress. In addition, several moderator variables that may potentially affect the size of the foreign genes effect on plant salt tolerance were also examined. A meta-analysis of the effect of *CPA1* overexpression has been previously published (Ma et al., 2017). Therefore, the main focus of the present study pertained to the overexpression of the *CIPK* and *CBL* gene family members. A meta-analysis was conducted on the impact of *CIPKs* and *CBLs* overexpression in transgenic plants subjected to salt stress across many studies. The effect of experimental variables on the impact of *CBL or CIPK* overexpression was also examined, and differences between *CBL* and *CIPK* effects on plants under salt-stress and non-stress conditions was also clarified. The results of the meta-analysis enabled us to identify areas of research requiring further investigation and increased our understanding of the function of *CBLs or CIPKs* in improving salt tolerance in plants.

## Materials and Methods

### Data Collection

Using the ISI Web of Science (Thompson Reuters; include 12 electronic databases), 633 unique articles on *CBL* and *CIPK* were located up through April, 2016. A total of 48 search terms, such as “*CBL*” “*CIPK*” AND (“overexpress” or “stress”), etc. were used to identify relevant studies (additional details provided in **Appendix S1**).

After closer examination, 591 of the articles were excluded because they did not conform to our inclusion standard. Reasons for exclusion included: data were unrelated to plants (421); data did not include the effect of *CBLs* or *CIPKs* overexpression on salt stress (108); they were review articles (12), they were patents (20) and figures or datasets (18); data were related but did not include the parameters on which we were focused (13), and; the same data had been reported in another article (1). This exclusion process resulted in retaining 40 articles for the meta-analysis. Of these, 15 articles pertained to the overexpression of *CBL* genes; 24 articles reported on the overexpression of *CIPK* genes, and only one article investigated the individual overexpression of both *CBL* and *CIPK* genes. The 39 articles were written in both English and Chinese (details provided in **Appendix S1**).

Treatment and control means, with sample sizes, were obtained for each study. If the sample size was given as a range, the smallest value in the range was used. If sample size was not reported, we defined it as *n* = 1 when no mean statistics were provided, or as *n* = 3 when standard errors or an LSD were provided. If the study included data from multiple time points, only the final time point was used in the meta-analysis. When the data were only presented in graphs, the numerical data was extracted using GetData Graph Digitizer^1^ software.

If multiple treatments were used in one article, each treatment was used as an independent study and represented as an individual unit in the meta-analysis. For example, He et al. (2013) examined both transformed and non-transformed *Arabidopsis* plants subjected to four different NaCl concentrations, which was represented as four individual studies in the meta-analysis. Hu et al. (2016) used two kinds of media in their study, which was then treated as two studies in the meta-analysis. Although the approach of breaking down a single study into multiple studies and designating them as independent studies may potentially result in an increase in the disadvantage of the statistical dependence among studies, which in the meta-analysis are assumed to be independent (Gurevitch and Hedges, 1999), the larger number of studies maximizes the statistical power of the meta-analysis (Lajeunesse and Forbes, 2003). Importantly, this approach has been commonly used in meta-analyses of plant biology (Holmgren et al., 2013; Klümper and Qaim, 2014), especially when the meta-analysis includes the evaluation of moderators (experimental variables) (Lehmann and Rillig, 2015).

### Effect Size and Moderators

**FIGURE 1:**
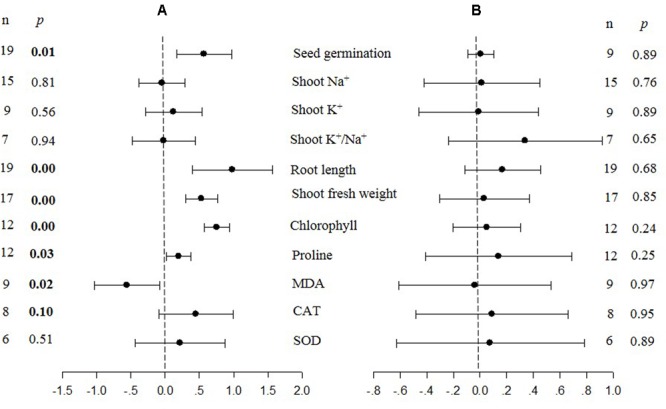
**Weighted summary effect sizes (ln *R*) and 95% confidence intervals (CIs) for the effect of CIPK overexpression in transgenic plants subjected to NaCl stress conditions **(A)** and non-stress conditions **(B)**.** A *p* ≤ 0.05 indicates that the moderator level was significantly different than zero; *n* stands for the number of studies.

**FIGURE 2:**
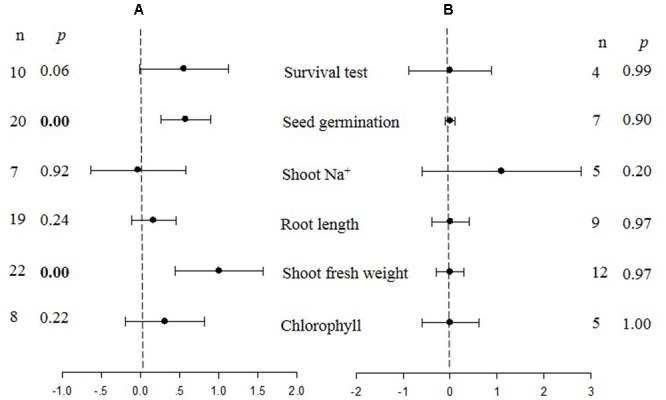
**Weighted summary effect sizes (ln *R*) and 95% CIs for the effect of CBL overexpression in transgenic plants subjected to NaCl stress conditions (A)** and non-stress conditions **(B)**. A *p* ≤ 0.05 indicates that the moderator level was significantly different than zero; *n* stands for the number of studies.

Each response variable was incorporated into the analysis as the ratio of transgenic to non-transgenic (control) plant means. This response ratio is referred to as the effect size, and its natural logarithm, ln *R*, is used in the meta-analysis (Borenstein et al., 2009), in the equation:

$$\begin{array}{c} \ln R=\ln Y_{\mathrm{TC}}/Y_{\mathrm{NC}} \end{array}$$

where *Y*_TC_ and *Y*_NC_ are the means of plants that were transformed (*TC*), or non-transformed (*NC*) (or transformed with an empty vector), with a *CBL* gene (**Figure 1**) or a *CIPK* gene (**Figure 2**), respectively. Response ratios refer to a standardized, unit-less expression of treatment-induced changes, that are commonly used in meta-analyses of plant response (Schaeffer et al., 2013; Worchel et al., 2013). The log transformation balances positive and negative treatment effects across response ratios and maintains symmetry in the analysis (Borenstein et al., 2009). In other words, summary effect sizes within a meta-analysis indicates the “response ratio” of transformed (TC)/non-transformed (NC) plants over all studies, and the natural logs of the response ratio reflects the relative magnitude of the treatment effect.

When Ln *R*-values are above 0, this indicates a TC-induced increase in the parameter, while values below 0 indicate a TC-induced decrease in the parameter; and a value of 0 signifies a lack of an effect by the overexpression of the transgene on that parameter.

Among the 39 articles, the only parameters that were selected for the determination of the summary effect size were those for which at least five studies from more than one article existed. On this basis, 11 summary effect sizes were computed for *CIPK* overexpression and 6 summary effect sizes were computed for *CBL* overexpression.

In addition to effect size, data for eight experimental attributes or “moderators” were collected from each study that may have impacted the response to salinity stress in the transgenic plants. Moderators were used to test for heterogeneity within effect sizes and were of two varieties: (1) experimental conditions: promoter, treatment medium, stress severity, and stress duration; (2) experimental materials: type (monocot vs. dicot), genus of gene donor and recipient, and whether the genera for the donor/recipient combination were the same or different. Each moderator included at least two levels (categories). The definition criteria for the moderators and categories were based on defined categories that were previously used in our laboratory (Ma et al., 2017).

In some cases, one experiment contained more than one level of salt stress severity. Therefore, we defined stress severity in three levels “low,” “medium,” and “high,” in order to evaluate whether or not the summary effects were impacted by different levels of stress severity. For example, when an experiment utilized 50, 100, 150, and 200 mM NaCl to expose plants to salt stress, we defined the 50 mM treatment as low, the 200 mM treatment as high, and 100 and 150 mM treatments as medium. Details on the defined and selected moderators are provided in **Appendix S1**.

### Meta-Analysis

Meta-analyses were conducted using Comprehensive Meta-Analysis (CMA) v.3 (Biostat, Englewood, NJ, United States; 2014) software and the weight of individual studies was carried out using non-parameter variance:

$$\begin{array}{c} V\ln R=(n_{\mathrm{TC}}+n_{\mathrm{NC}})/(n_{\mathrm{TC}}*n_{\mathrm{NC}}) \end{array}$$

where *V*ln *R* is the variance of the natural log of the response ratio *R* and *n*_TC_ and *n*_NC_ are the sample sizes of the TC and NC treatments (Borenstein et al., 2009). A detailed description of meta-analysis was previously described (Ma et al., 2017).

### Heterogeneity

The *Q* statistic, which is based on weighted squared deviations, was used to analyze the heterogeneity and *I*^2^, which is a descriptive index that estimates the ratio of true heterogeneity to total heterogeneity across the observed effect sizes (Borenstein et al., 2009). In other words, the *Q* statistic was used to quantify heterogeneity.

### Publication Bias

Studies that report relatively high effect sizes are more likely to be published than studies that report lower effect sizes. In the meta-analysis, any bias in the literature is likely to be reflected. This issue is known as publication bias (Borenstein et al., 2009). Potential publication bias was statistically examined using three kinds of general techniques designed to evaluate publication bias. These were the symmetry/asymmetry of a funnel plot (Viechtbauer, 2007), the Begg and Mazumdar rank (Kendall) correlation (Begg and Mazumdar, 1994; Borenstein et al., 2009), and the Egger’s linear regression method (Sterne and Egger, 2005).

## Results

### Summary Effects

Eleven different species (**Appendix S1**) served as gene donor plants among the 125 studies conducted on *CIPK*-overexpression. The most widely used *CIPK* genes were obtained from *Populus* (35 studies), and the majority (87 studies) of gene donor plants were dicotyledons. There were eight different genera of recipient plants, with the majority of recipient plants being represented by *Arabidopsis* (54 studies) and the overall majority of recipient plants were dicots (117 studies). The data set obtained from the 125 studies included the use of three different promoters, with the *35s* promoter being the most utilized (115 studies), followed by *Ubi* (8 studies), and lastly rd29A (2 studies).

Seven plant species served as gene donor plants among the 97 studies conducted on *CBL*-overexpression in plants (**Appendix S1**). Similar to *CIPK* studies, *Populus* was the most highly represented donor of *CBL* genes (39 studies), and dicots were the most highly represented among all the *CBL* donor species (93 studies). Gene recipient plants were represented by three species, with *Arabidopsis* being the most highly represented (95 studies). The *CBL* data set included the use of two promoters, with *35s* being used most often (91 studies), followed by *rd29A* (2 studies). Four of the studies did not include the type of promoter that was used to drive *CBL* expression.

**FIGURE 3:**
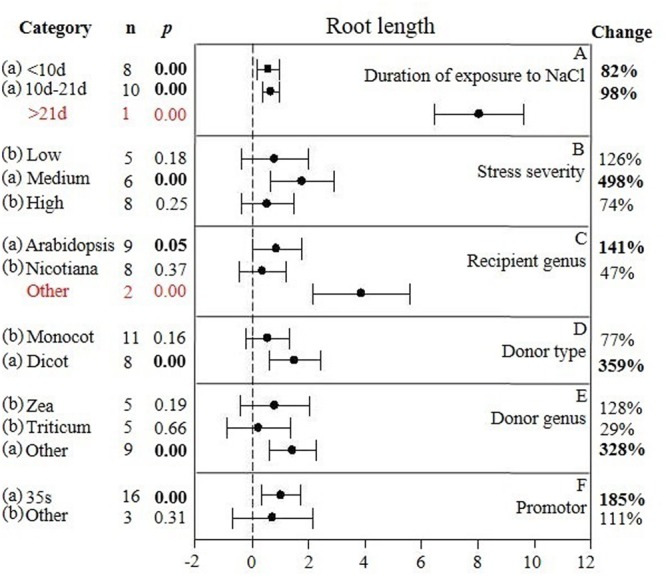
**Weighted summary effect sizes (ln *R*) and 95% CIs showing how moderator variables affect the extent to which CIPK transformation modifies root length. (A)** Duration of exposure to NaCl, **(B)** Stress severity, **(C)** Recipient genus, **(D)** Donor type, **(E)** Donor genus, and **(F)** Promotor. A *p* ≤ 0.05 indicates that the moderator level was significantly different than zero; *n* stands for the number of studies.

**FIGURE 4:**
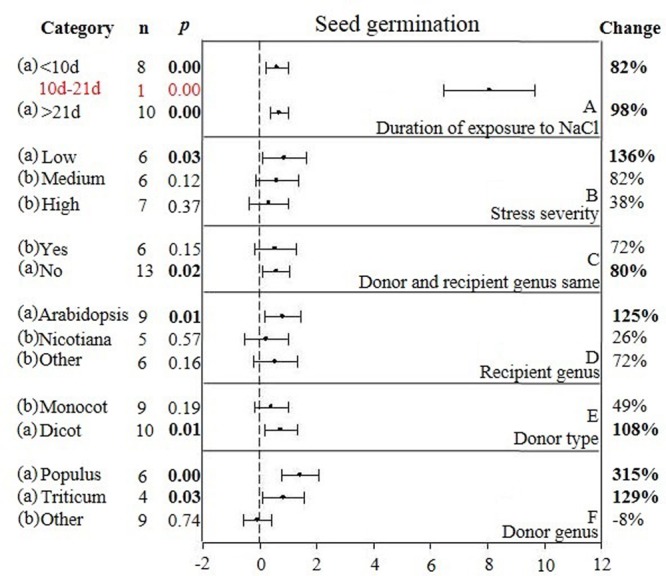
**Weighted summary effect sizes (ln *R*) and 95% CIs showing how moderator variables affect the extent to which CIPK transformation modifies seed germination. (A)** Duration of exposure to NaCl, **(B)** Stress severity, **(C)** Donor and recipient genus same, **(D)** Recipient genus, **(E)** Donor type, and **(F)** Donor genus. A *p* ≤ 0.05 indicates that the moderator level was significantly different than zero; *n* stands for the number of studies.

**FIGURE 5:**
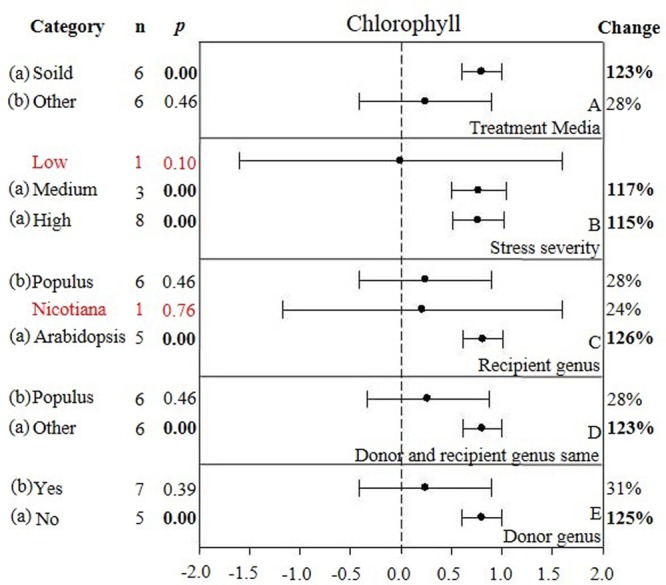
**Weighted summary effect sizes (ln *R*) and 95% CIs showing how moderator variables affect the extent to which CIPK transformation modifies chlorophyll. (A)** Treatment media, **(B)** Stress severity, **(C)** Recipient genus, **(D)** Donor and recipient genus same, and **(E)** Donor genus. A *p* ≤ 0.05 indicates that the moderator level was significantly different than zero; *n* stands for the number of studies.

**FIGURE 6:**
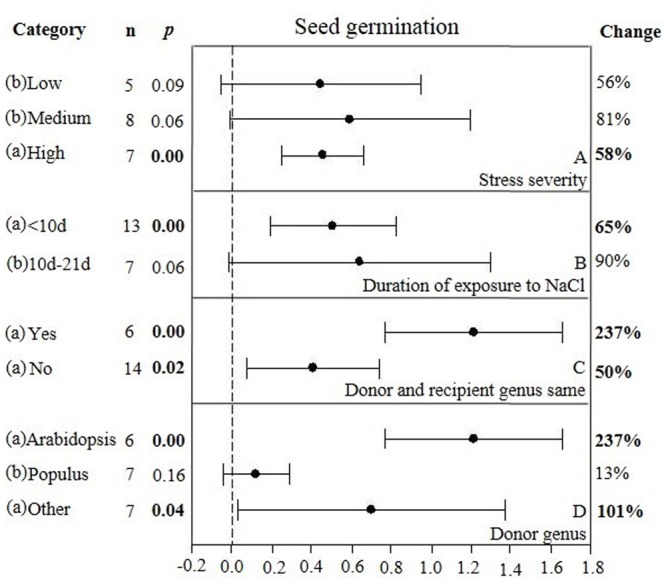
**Weighted summary effect sizes (ln *R*) and 95% CIs showing how moderator variables affect the extent to which CBL transformation modifies seed germination. (A)** Stress severity, **(B)** Duration of exposure to NaCl, **(C)** Donor and recipient genus same, and **(D)** Donor genus. A *p* ≤ 0.05 indicates that the moderator level was significantly different than zero; *n* stands for the number of studies.

**FIGURE 7:**
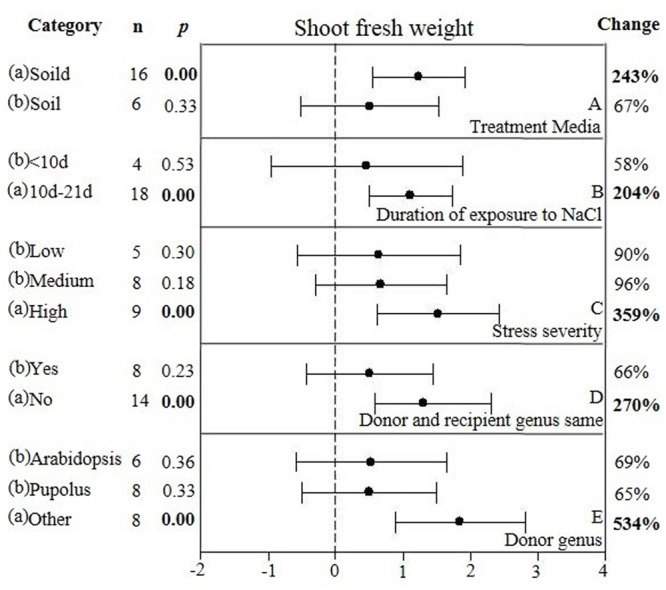
**Weighted summary effect sizes (ln *R*) and 95% CIs showing how moderator variables affect the extent to which CBL transformation modifies shoot fresh weight. (A)** Treatment media, **(B)** Duration of exposure to NaCl, **(C)** Stress severity, **(D)** Donor and recipient genus same, and **(E)** Donor genus. A *p* ≤ 0.05 indicates that the moderator level was significantly different than zero; *n* stands for the number of studies.

**Figures 1**–**7** and **Supplementary Figures S1**–**S3** are forest plots that illustrate the summary (main) effects of *CIPK* and *CBL* overexpression, in transgenic plants, relative to non-transgenic plants, subjected to non-stress and NaCl stress conditions. The relative magnitude of each treatment effect and their CIs are presented in the forest plots, in which *Ln R*-values of the summary effect above 0 indicate that *CIPK* or *CBL* overexpression had a positive effect (increased) on that attribute. Values below 0 indicate that the overexpression had a negative (decreased) effect on that attribute. Summary effects are considered significant if their 95% CIs did not overlap zero. The raw percentage of the change resulting from either *CIPK or CBL* overexpression are listed in **Tables 1, 2**, respectively.

**Table 1:** Heterogeneity statistics for the 11 summary effect sizes under NaCl stress.

Summary effect size	*Qt*	*p*_-hetero_	*I^2^* (%)	TC-induced change (%)
Seed germination	22.774	0.199	21	**77**
Shoot Na^+^	3.987	0.996	0.0	–4
Shoot K^+^	0.549	1.000	0.0	13
Shoot K^+^/Na^+^ ratio	1.179	0.978	0.0	–2
Root length	29.332	0.045	39	**168**
Shoot fresh weight	6.67	0.979	0.0	**70**
Chlorophyll	5.024	0.930	0.0	**114**
Proline	2.337	0.997	0.0	**22**
MDA	7.795	0.454	0.0	–**43**
CAT	2.054	0.957	0.0	57
SOD	0.301	1.000	0.0	25

*Qt, total heterogeneity; *p*_*-hetero*_, probability that *Qt* was due entirely to sampling error and not to variation among true effects; *I*^*2*^, percentage of heterogeneity due to variation among true effects; changes to summary effects caused by foreign *CIPK* genes, transformed from ln R to raw percentages. For TC-induced changes, bold text signifies change was significant (*p* ≤ 0), with positive values indicating TC-induced promotion and negative values TC-induced inhibition.*

**Table 2:** Heterogeneity statistics for the six summary effect sizes under NaCl stress.

Summary effect size	*Qt*	*p*_-hetero_	*I*^2^ (%)	TC-induced change (%)
Survival test	9	0.342	0.0	74
Seed germination	21.528	0.308	11.743	**78**
Shoot Na^+^	1.311	0.971	0.0	–3
Root length	4.508	0.999	0.0	18
Shoot fresh weight	37.433	0.015	43.899	**173**
Chlorophyll	0.583	0.999	0.0	37

*Qt, total heterogeneity; *p*_*-hetero*_, probability that *Qt* was due entirely to sampling error and not to variation among true effects; *I*^*2*^, percentage of heterogeneity due to variation among true effects; changes to summary effects caused by foreign *CBL* genes, transformed from ln R to raw percentages. For TC-induced changes, bold text signifies change was significant (*p* ≤ 0), with positive values indicating TC-induced promotion and negative values TC-induced inhibition.*

The meta-analysis indicated that six plant parameters were significantly impacted by foreign *CIPK* overexpression in plants subjected to NaCl stress (**Figure 1A**: *p* ≤ 0.05). Among the six parameters, root length exhibited the largest increase, with an approximately 2.7× higher increase in transgenic (TC) plants, relative to (NC) plants, subjected to NaCl conditions. Chlorophyll levels also increased dramatically by 114% in TC plants, relative to their NC counterparts. Shoot fresh weight and seed germination were also markedly higher in TC plants. Overexpression of *CIPK* genes resulted in a significant reduction of the MDA content in leaves. Shoot Na^+^ and shoot K^+^/Na^+^ were reduced by 2 to 4%, although this was not considered significant since the 95% CI overlapped with zero. In contrast to NaCl stress conditions, the overexpression of *CIPK* genes did not significantly impact (*p* ≥ 0.05) any of the plant parameters in transgenic plants subjected to non-stress conditions (**Figure 1B**).

Only two summary effects (TC/NC response ratios) were significantly impacted in transgenic plants overexpressing a *CBL* gene when they were exposed to NaCl stress (**Figure 2A**: *p* ≤ 0.05). Shoot fresh weight exhibited a 173% increase in TC plants, relative to NC plants, subjected to NaCl stress, and seed germination increased by 78% in TC plants, relative to NC plants. None of the impacts on the summary effects were significant under non-stressed conditions (**Figure 2B**).

Probability (*p*) values for the summary effects directly reflect the magnitude of the significance of the treatment effect, but may also indirectly convey information about the availability (sample size) on which the summary effect is based on (Borenstein et al., 2009). Large but insignificant summary effects may reflect real treatment differences for which sample sizes are simply insufficient to account for the variability present in the data set (i.e., statistical power is too low to reveal significance). For example, this may be the case for CAT and SOD in *CIPK* overexpressing plants subjected to NaCl stress (**Figure 1A**), and root length in unstressed plants (**Figure 2B**). Therefore, more attention should be placed on the magnitude and precision of the summary effects in a meta-analysis than on tests of their statistical significance or the *p*-values (Cooper, 2009; Cumming, 2012).

### Publication Bias

Based on the parameters that are commonly used to test for publication bias in meta-analysis, no evidence of publication bias was found (**Supplementary Tables S1, S2**). The Begg and Mazumdar rank correlation test derives the rank correlation between standardized effect sizes and their standard errors (estimated from their non-parametric variances) (Begg and Mazumdar, 1994). Results indicated that most summary effects had a *p* > 0.05, indicating little concern for bias. Across summary effects in our study, most of the funnel plots across summary effects were symmetric and also raised no concerns about publication bias (**Supplementary Tables S1, S2**). Kendall tau values were also below 0.3 for all of the summary effects with *p* > 0.05, indicating little evidence for bias. A two-tailed significance test (Egger’s *p*-values) in *CIPK* overexpressing plants suggested the possibility of a publication bias for shoot Na^+^, and MDA. The two-tailed significance tests (Egger’s *p*-values) in *CBL* overexpressing plants, however, suggested little concern for publication bias.

### Heterogeneity and Moderator Analysis

Heterogeneity represents true differences in treatment outcomes among studies. Moderator analyses are conducted to reveal heterogeneity and to determine which experimental variables (moderators) significantly influenced treatment outcomes and which have had little or no effect. A *P-*_hetero_ value for a *Q*-test < 0.1 or a positive *I*^2^ was used to indicate significant heterogeneity in the size of the summary effect. A *p-*_hetero_ value for a *Q*-test > 0.1 indicates that the impact of the moderator on the summary effect is inconsistent among studies (Iacovelli et al., 2014). Due to the low statistical power brought about by a small number of available studies for a particular parameter or large within-study variance, the substantial real dispersion of true effects might frequently yield a *p-*_hetero_ > 0.1. This situation often arises in meta-analyses of plant studies. As a result, although only two of the summary effects in *CIPK* overexpressing plants subjected to NaCl stress exhibited significant heterogeneity (**Table 1**, root length: *p-*_hetero_ values less 0.10, and *I*^2^ = 39; seed germination: *p-*_hetero_ values exceeded 0.10, however, *I*^2^ = 21), and two summary effects in *CBL* overexpressing plants subjected to NaCl stress exhibited statistically significant heterogeneity (**Table 2**, seed germination*: p-*_hetero_ values exceeded 0.10, however, *I*^2^ = 11.743; Shoot fresh weight: *p-*_hetero_ values less 0.10, and *I*^2^ = 43.899), a moderator analysis on all summary effects which was affected significant by foreign *CIPK* genes or *CBL* genes was still performed. Results of the meta-analysis indicated that six summary effects were significantly affected by *CIPK* transformation (**Figure 1A**) and two were significantly affected by *CBL* transformation (**Figure 2A**). Therefore, a moderator analysis on all eight summary effects was conducted and the results are displayed in **Figures 3**–**7** and **Supplementary Figures S1**–**S3**. The results of the analysis revealed the influence of the moderator categories on the summary effects and indicated which summary effect was most affected by a moderator. If the influence of a moderator category has a significant impact due to overexpression, it is marked with an “a.” If the influence of a moderator category is not significant, it is marked with a “b.” If the sample size of a moderator category is too small (*n* < 3) to have the statistical significance, it was marked in red (**Figures 3**–**7**).

The individual impact of several different moderators on the degree to which *CIPK* overexpression affected root length in salt-stressed plants is presented in **Figure 3**. There were two of the six moderators significantly affected the TC-induced increase in root length observed in *CIPK*-overexpressing plants subjected to NaCl stress. Due to the small sample size (*n* < 3), the duration of the exposure to salt stress only had a minor effect on the TC-induced increase in root length, with the exception at the longest durations (more than 21 days) (**Figure 3A**). An increase in the severity of the salt stress from low to medium, or a reduction from high to medium stress levels resulted in a 4× or 7× increase in the TC-induced impact on root length, respectively (**Figure 3B**). When the gene recipient was *Arabidopsis*, it was apparent that root length was more responsive to *CIPK* overexpression than *Nicotiana*. However, even though the effect of other recipient genera appeared to be quite large, it was not statistically significant due to the small sample size (**Figure 3C**). The general category of donor type (monocot vs. dicot) greatly affected the magnitude of the TC-induced increase in root length when the donor was a dicot, with the impact being 4× greater than when the donor was a monocot (**Figure 3D**). As a gene donor genus, although *Triticum* had only a modest effect on the TC-induced increase in root length in plants subjected to salt stress, increasing the *CIPK*-overexpression effect by 29%; compared to a 128% increase in root length when the gene donor genus was *Zea*. The effect of *Triticum* and *Zea* as *CIPK* gene donors, however, was no significant impact after overexpression and was less than when the genus of the gene donor was other than these two genera (**Figure 3E**). The type of promoter used to overexpress *CIPK* had little effect on the TC-induced effect on root length in plants subjected to NaCl stress (**Figure 3F**).

Two of the six moderators significantly affected the TC-induced increase in seed germination observed in *CIPK*-overexpressing plants subjected to NaCl stress (**Figure 4, Table 3**, and **Appendix S1**). Similar to root length, the lowest (<10 days) and highest (>21 days) durations of salt stress had little impact on the TC-induced increase in seed germination (**Figure 4A**). The effect of an intermediate duration (10–21 days) was not included in the analysis due to insufficient sample size (*n* < 3). The effect of salt stress severity on TC-induced increases in seed germination was greatest at low levels of severity, followed by medium and then high levels of severity (**Figure 4B**). The influence of the donor/recipient combination on seed germination in *CIPK*-overexpressing plants subjected to salt stress was very similar, regardless if the donor/recipient were different genera or the same genera (**Figure 4C**). The influence of just the recipient genus on the TC-induced increase in seed germination in NaCl stressed plants was much greater when *Arabidopsis* was the recipient genus as compared to when *Nicotiana* and other genera were recipients (**Figure 4D**). Similar to root length, the TC-induced increases in seed germination was not affected by the general category of donor type (**Figure 4E**), though the more specific moderator of donor genus had a strong and statistically significant effect on the TC-induced increase in seed germination (**Figure 4F**). When the donor genus was *Populus*, the average increase was 315%, 129% when it was *Triticum*, and an 8% decrease, relative to non-transgenic plants when the gene donor genus was some other genus.

**Table 3:** Categorical moderators examined for the six summary effects of CIPK-overexpression that were significantly different from zero Heterogeneity *p*-values (*p-*_hetero_) are shown here.

Moderator	Effect size				
	Seed germination	Root length	Shoot fresh weight	Chlorophyll	Proline	MDA
Treatment media	–	–	–	0.109	0.520	0.180
Duration of exposure to NaCl	0.000	0.000	0.627	–	0.545	0.633
Stress severity	0.598	0.245	0.815	0.643	0.691	–
Recipient genus	0.524	0.001	0.616	0.194	–	0.320
Donor type	0.423	0.117	0.470	–	–	–
Donor genus	0.000	0.235	0.396	0.109	0.740	0.320
Donor and recipient genus same	0.920	–	0.950	0.095	0.975	0.179
Promoter	–	0.711	–	–	–	–

*probability that all observed (total) heterogeneity is not due solely to sampling error (expected variation) *p*_*-hetero*_ ≤ 0.1 indicates significant heterogeneity among true effects. Dashed entries signify that insufficient data were available to perform an analysis. See supplement for associated *Qm* values (between study heterogeneity); *n* (sample size); *df* (degrees of freedom, levels within a moderator).*

Only one of the five moderators significantly affected the TC-induced increase in chlorophyll observed in *CIPK*-overexpressing plants subjected to NaCl stress (**Figure 5, Table 3**, and **Appendix S1**). The effect of *CIPK* overexpression on chlorophyll content in salt-stressed plants was approximately twice as large (123%) when plants were grown on other media (28%) (**Figure 5A**). The severity of the stress severity had little differential impact on the TC-induced effect on chlorophyll content, with the exception at the low severity level, which was not considered due to a small sample size (*n* < 3). As with the previous summary effect, the moderator influence was the greatest when the recipient genus was *Arabidopsis* (125%) (**Figure 5C**). The TC-induced effect on chlorophyll content was greater when the donor genus was in the ‘other’ category as compared to when the donor genus was *Populus* (**Figure 5D**). The impact of *CIPK-*overexpression on chlorophyll content was greater when the donor/recipient combination was composed of different genera than when they were composed of the same genera (**Figure 5E**).

There were two of the four moderators significantly affected the TC-induced increase in seed germination observed in *CBL*-overexpressing plants subjected to NaCl stress (**Figure 6, Table 4**, and **Appendix S1**). The level of stress severity (**Figure 6A**), and variations in the duration of stress exposure (**Figure 6B**), had differential impact on the extent to which the overexpression of *CBL* overexpression in transgenic plants exposed to salt stress increased seed germination. The TC-induced effect of *CBL* overexpression on seed germination when the donor/recipient combination was composed of different genera was only a quarter of what it was when the donor/recipient combination was composed of the same genera (51% vs. 238%, respectively) (238% vs. 51%) (**Figure 6C**). TC-induced enhancement of seed germination in salt stressed plants was significantly affected by the donor genus (**Figure 6D**). A much higher level of germination was observed when the genus of the gene donor was *Arabidopsis* (238%), as compared to when it was *Populus* (13%) or others (101%).

**Table 4:** Categorical moderators examined for the six summary effects of *CBL*- transformation that were significantly different from zero Heterogeneity *p*-values (*p*-_hetero_) are shown here.

Effect size	Moderator				
	Treatment media	Duration of exposure to NaCl	Stress severity	Donor and recipient genus same	Donor genus
Seed germination	–	0.716	0.915	0.005	0.000
Shoot fresh weight	0.254	0.412	0.365	0.190	0.100

*probability that all observed (total) heterogeneity is not due solely to sampling error (expected variation) *p*_*-hetero*_ ≤ 0.1 indicates significant heterogeneity among true effects. Dashed entries signify that insufficient data were available to perform an analysis. See supplement for associated *Qm* values (between study heterogeneity); *n* (sample size); *df* (degrees of freedom, levels within a moderator).*

Only one of the five moderators significantly affected the TC-induced increase in fresh weight observed in *CBL*-overexpressing plants subjected to NaCl stress (**Figure 7, Table 4**, and **Appendix S1**). *CBL* overexpression had approximately 4× the effect on increased shoot fresh weight when plants were grown on solid media (236%), as compared to soil grown plants (66%) (**Figure 7A**). TC-induced increases in shoot fresh weight were greater when the duration of the stress was 10–21 days, as compared to when the stress was applied for shorter durations (**Figure 7B**). Increases in shoot fresh weight were significantly greater (358%) at the highest level of stress severity than it was at moderate and low levels of salt stress severity (90 and 96%, respectively) (**Figure 7C**). Whether the donor and recipient were of the same genus, had little influence on the TC-induced increase in shoot fresh weight, and of different genera, had significant influence on the TC-induced increase in shoot fresh weight in *CBL*-overexpressing plants subjected to NaCl stress (**Figure 7D**). The TC-induced increase in shoot fresh weight was significantly affected by the genus of the donor (**Figure 7E**). The average increase in shoot fresh weight was 69% when the donor genus was *Arabidopsis*, 65% when the donor genus was *Populus*, and 534% when the donor was a genus other than the *Arabidopsis* or *Populus*.

## Discussion

The CBL-CIPK signaling network has been demonstrated to be involved in plant responses to salt stress, in which the salt-overly-sensitive (SOS) pathway is a classic. The SOS pathway is comprised of three key components: *SOS1/NHX7, SOS2/CIPK24*, and *SOS3/CBL4* (Zhu, 2002). It has been reported that overexpression of CBL-CIPK network genes in plants could increase their ability to maintain ionic homeostasis and thus increase salt tolerance (**Appendix S1**). Numerous studies have been conducted in the past decade to overexpress native or ectopic *CBL* and *CIPK* genes in a variety of crop species in an attempt to develop transgenic salt-tolerant crops (**Appendix S1**). In the present study, a meta-analysis of published data was conducted in order to comprehensively determine the effect of overexpressing either *CBL* or *CIPK* genes on various plant parameters in transgenic plants subjected to salt stress, as well as to identify the moderators (experimental variables) that influence the impact of *CBL* or *CIPK* overexpression on those parameters.

### Salt Tolerance Related Traits That Were Significantly Improved by the Overexpression of CBL-CIPK Genes in Transgenic Plants

Over-expression of *CIPK* gene family members significantly improves five salt-tolerance related plant parameters and decreases one parameter (**Figure 1A**). One of the parameters that was most significantly improved is root length, which was 168% greater in transgenic plants exposed to NaCl stress, as compared to non-transgenic plants. Another major effect was an increase in chlorophyll content, which exhibited an average increase of 114%. The higher levels of chlorophyll in transgenic plants subjected to salt stress would contribute to their ability to sustain photosynthesis and assimilation during episodes of salt stress (Smillie and Nott, 1982; Rout et al., 1997). The overexpression of *CIPK* genes in transgenic plants also resulted in a 77% increase in seed germination when the transgenic plants were subjected to salt stress, a reliable indicator of salt tolerance (Tahal et al., 2000; Shabala and Cuin, 2008). It is worth noting that the overexpression of *CIPK* gene family members had no significant effect on these plant parameters in plants growing under non-stress conditions (**Figure 1B**). Overexpression of *CBL* genes significantly improved just two plant salt-tolerance related traits in transgenic plants subjected to salt stress (**Figure 2A**). One of the most significant effects was a 173% improvement in the shoot fresh weight of transgenic plants, while the other major effect was a 78% increase in seed germination. No significant effect on these traits was observed when plants were grown under non-stress conditions (**Figure 2B**). The results of the meta-analysis clearly demonstrate that overexpression of *CBL* or *CIPK* family member genes can improve the performance of plants when they are subjected to salt stress. When plants are subjected to abiotic stress, a stress signal is generated that specifically involves Ca^2+^ (Rudd and Franklin-Tong, 2001; Sanders et al., 2002; Dodd et al., 2010). Previous studies have demonstrated that the interaction between *CBL* and *CIPK* requires the micromolar levels of Ca^2+^ (Halfter et al., 2000; Akaboshi et al., 2008). The level of Ca^2+^ required for the CBL-CIPK complex to be activated can vary depending on the participating *CBL* and *CIPK* members, but is still nevertheless required for activation. Ca^2+^ signals are elicited by many diverse stimuli, including salinity, and the CBL-CIPK system is involved in transmitting the stress perception via the Ca^2+^ signaling pathway (Yu et al., 2014; Zhou et al., 2014). It is plausible that this might be the reason why overexpression of the *CBL* and *CIPK* gene family members had no effect on the measured salt-tolerance related plant parameters in the absence of salt stress; since salt stress changes the Ca^2+^ concentration in plants and the CBL-CIPK complex requires Ca^2+^ to become activated.

### The Influence of Experimental Variables (Moderators) on *CIPK* or *CBL* Induced Changes in Transgenic Plants Subjected to Salt Stress

The donor genus had a significant influence on the CIPK and CBL overexpression effect on seed germination. Previous studies have demonstrated that the CBL-CIPK complex acts as a negative regulator of the ABA signaling during seed germination (Kim et al., 2003; Pandey et al., 2004, 2008), however, *CBL* is a positive regulator of the GA pathway (Hwang et al., 2005). Therefore, we suggest, based on the meta-analysis, that *CIPK* and *CBL* differ in their regulatory function and that the regulatory mechanisms associated with these genes are genus-specific and perhaps gene family member specific. In this regard, seed germination was the greatest when the *CIPK* gene was obtained from *Populus* (315%) and the *CBL* gene was obtained from *Arabidopsis* (237%). Shoot fresh weight in transgenic plants overexpressing either *CIPK* or *CBL* genes exhibited a significant increase, relative to non-transgenic plants, when plants were grown under salt stress. The potential role of the CBL-CIPK system in inorganic nutrient sensing (Cheong et al., 2003, 2007; Xu et al., 2006; Hu et al., 2009), may explain these results. The size of the effect on shoot fresh weight, however, was affected by the donor genus in *CBL* transgenic plants. Therefore, we suggest that overexpression of *CBL* or *CIPK* genes can improve the shoot fresh weight of plants grown under salt stress conditions, but that the extent of the improvement is dependent on the binding capacity of *CBL* to stress signaling components; a CBL characteristic that is genus-specific. Further research will be required to determine how the *CBLs* from different genera differ in their ability to affect shoot fresh weight.

The meta-analysis indicated that gene donor/recipient combinations had a significant moderating influence on the *CBL* or *CIPK* induced increase in seed germination and chlorophyll content, respectively. When the genera of the *CBL* gene donor/recipient combination were different, seed germination was greater than when the genera of the *CBL* gene donor/recipient combination were the same. These results suggest that when studies are conducted on CBL-CIPK as a complex or a system in their response to abiotic stress (Xu et al., 2006; Pandey et al., 2008), that the impact of overexpression on stress tolerance related parameters may vary depending on the genus from which the *CIPK* or *CBL* gene is obtained; as well as the genus of the recipient plant.

### Future Research

Salt stress induces many physiological changes in plants and numerous alterations in many parameters have been documented, including the compatible solutes, relative electrolyte leakage (REL), and K^+^ and other ionic balances (Ma et al., 2014; Song et al., 2014). Many of these parameters, however, were not measured in the available *CIPK* or *CBL* overexpression studies. Therefore, these parameters were not included in our meta-analysis due to small sample size (*n* < 5). Many of the classic physiological changes that occur in plants in response to salt stress were rarely examined in *CBL* and *CIPK* overexpression studies. As a result, it is unclear whether or not these parameters would even be affected in the transgenic plants, or what role they might play in salt response/tolerance.

Ca^2+^ has been identified as a key signaling molecule (Ditta, 2013), and the CBL-CIPK system has been identified as a calcium dependent signaling pathway. Few studies of *CBL* or *CIPK* -transformed plants, however, have included measurements of Ca^2+^ concentration in plant tissues. Previous studies have also shown that the CBL-CIPK network regulates an inward rectifier K^+^ channel, such as *AKT1* (Li et al., 2006; Cheong et al., 2007), and regulates the high-affinity K^+^ transporter, such as *AtHAK5* (Ragel et al., 2015). Again, however, K^+^ concentration in the root or in shoots has rarely been measured (*n* < 5) in *CBL* and *CIPK*-overexpressing plants. Measurement of K^+^ and Ca^2+^ changes in future studies would contribute greatly to developing a better understanding of salt tolerance in relation to the role of CBL-CIPK. Almost all of the *CBL* genes in the transgenic studies have been obtained from dicots. In fact, only four studies used monocots as the source of the transgene. The majority of recipient plants have also been dicots, with only two studies in one report using a monocot as a recipient. Therefore, it remains unclear whether or not *CBL* genes from monocotyledonous plants, when over-expressed in monocots, will improve salt tolerance as markedly as they do in dicots. A similar situation exists for the use of *CIPK* genes. Among 125 studies, only 8 studies overexpressed the *CIPK* gene in monocotyledonous plants. Therefore, it still remains unclear whether or not *CIPK* overexpression in monocots definitively improves salt tolerance.

Experimental evidence has suggested that *CIPK* genes are activated by *CBL* genes in a Ca^2+^-dependent manner (Kim et al., 2003) and that the CBL-CIPK complex post-translationally phosphorylates downstream target proteins (Xu et al., 2006). On this basis, one would predict that overexpressing both *CBL* and *CIPK* genes together in one recipient plant would be more effective in promoting salt tolerance than the overexpression of just a single one of these genes. Unfortunately, this approach was only used in two studies and, therefore, could not be used as a moderator in our meta-analysis due to the small sample size. As more studies using this approach are conducted, it would be interesting to include this approach as a moderator in future meta-analyses to better understand the effect of *CIPK* and *CBL* overexpression.

## Author Contributions

YM, QC, and ZC designed the experiment. YM, QC, and HL performed the experiment. YM, JL, and YC analyzed the data. YM and ZC wrote the paper. All of the authors read and approved the final manuscript.

## Conflict of Interest Statement

The authors declare that the research was conducted in the absence of any commercial or financial relationships that could be construed as a potential conflict of interest.

## Footnotes

1 http://getdata-graph-digitizer.com

## Supplementary Material

APPENDIX S1 **Details of all the paper, key word, effect size and mordoritor**.

TABLE S1 **Measures used in characterizing publication bias for each effect size of CIPK transformation**.

TABLE S2 **Measures used in characterizing publication bias for each effect size of CBL transformation**.

FIGURE S1 **Weighted summary effect sizes (ln *R*) and 95% confidence intervals (CIs) showing how moderator variables affect the extent to which CIPK transformation modifies shoot fresh weight.** A *p* ≤ 0.05 indicates that the moderator level was significantly different than zero; *n* stands for the number of studies.

FIGURE S2 **Weighted summary effect sizes (ln *R*) and 95% CIs showing how moderator variables affect the extent to which CIPK transformation modifies MDA levels.** A *p* ≤ 0.05 indicates that the moderator level was significantly different than zero; *n* stands for the number of studies.

FIGURE S3 **Weighted summary effect sizes (ln *R*) and 95% CIs showing how moderator variables affect the extent to which CIPK transformation modifies proline levels.** A *p* ≤ 0.05 indicates that the moderator level was significantly different than zero; *n* stands for the number of studies.
