# Supplementary material for: Identification of Important Physiological Traits and Moderators That Are Associated with Improved Salt Tolerance in CBL and CIPK Overexpressors through a Meta-Analysis
Source: Front Plant Sci. 2017 May 29;8:856. doi: 10.3389/fpls.2017.00856 (PMC5446987; doi:10.3389/fpls.2017.00856)
Supplement: TABLE S1 — Measures used in characterizing publication bias for each effect size of CIPK transformation. [file Table_1.docx]

**Table S1** Measures used in characterizing publication bias for each effect size of CIPK transformation

| Effect size | Summary effect^1^ | | | Funnel^2^ | Kendall^3^ | | Egger’s^4^ | | |
| --- | --- | --- | --- | --- | --- | --- | --- | --- | --- |
|  | N | ln*RR* | *p* | plot | tau | *p* | | *β* | *p* |
| Seed germination | 28 | 0.390 | 0.009 | yes | -0.01 | 0.95 | -1.18 | | 0.66 |
| shoot Na+ | 23 | -0.021 | 0.876 | no | -0.28 | 0.06 | -1.88 | | <0.001 |
| Shoot K+ | 16 | 0.063 | 0.686 | no | 0.20 | 0.28 | -0.14 | | 062 |
| Shoot K+/Na+ ratio | 13 | 0.121 | 0.510 | no | -0.05 | 0.81 | 0.73 | | 0.46 |
| Root length | 29 | 0.664 | 0.001 | no | 0.17 | 0.21 | 1.26 | | 0.24 |
| Shoot fresh weight | 25 | 0.368 | 0.000 | no | 0.00 | 1.00 | -0.20 | | 0.69 |
| Chlorophyll | 20 | 0.481 | 0.000 | no | -0.03 | 0.85 | -0.62 | | 0.17 |
| Proline | 18 | 0.194 | 0.026 | no | 0.26 | 0.13 | 0.21 | | 0.21 |
| MDA | 16 | -0.354 | 0.055 | no | -0.05 | 0.79 | 4.09 | | <0.001 |
| CAT | 15 | 0.282 | 0.162 | no | 0.10 | 0.59 | 0.63 | | 0.75 |
| SOD | 11 | 0.154 | 0.530 | no | 0.05 | 0.81 | 2.13 | | 0.53 |

^1^Summary effect: n=number of studies, ln*RR* = natural log of overall summary effect, *p*= probability that summary effect ≠ 0

^2^Funnel plot appears asymmetrical

^3^Begg and Mazumdar Kendall rank correlation: tau = rank correlation coefficient (with continuity correction), two-tailed *p* = probability that study effect sizes are correlated with their sampling variances

^4^Egger’s linear regression: *β =* intercept of regression line, *p* = probability of significant asymmetry in study effect size/study size association. The regression runs through zero if the funnel plot is symmetrical. The size of the deviation of the intercept from the origin is a measure of asymmetry, with two-tailed *p* <0.05 indicating significant asymmetry (Sterne and Egger 2005)
