# Supplementary material for: Identification of Important Physiological Traits and Moderators That Are Associated with Improved Salt Tolerance in CBL and CIPK Overexpressors through a Meta-Analysis
Source: Front Plant Sci. 2017 May 29;8:856. doi: 10.3389/fpls.2017.00856 (PMC5446987; doi:10.3389/fpls.2017.00856)
Supplement: TABLE S2 — Measures used in characterizing publication bias for each effect size of CBL transformation. [file Table_2.docx]

**Table S2** Measures used in characterizing publication bias for each effect size of CBL transformation.

| **Effect size** | Summary effect^1^ | | | | Funnel^2^ | | Kendall^3^ | | | Egger’s^4^ | | |
| --- | --- | --- | --- | --- | --- | --- | --- | --- | --- | --- | --- | --- |
|  | N | ln*RR* | *p* | plot | | tau | | *p* | *β* | | | *p* |
| Survival test | 14 | 0.393 | 0.105 | no | | 0.08 | | 0.70 | -0.22 | | 0.80 | |
| Seed germination | 20 | 0.578 | 0.000 | no | | -0.08 | | 0.73 | -1.9 | | 0.47 | |
| Shoot Na+ | 12 | 0.469 | 0.242 | yes | | 0.20 | | 0.28 | -0.14 | | 062 | |
| Root length | 28 | 0.114 | 0.328 | no | | 0.21 | | 0.11 | 0.17 | | 0.55 | |
| Shoot fresh weight | 34 | 0.636 | 0.001 | no | | 0.25 | | 0.04 | 1.18 | | 0.22 | |
| Chlorophyll | 13 | 0.186 | 0.347 | no | | 0.00 | | 1.00 | -0.37 | | 0.50 | |

^1^Summary effect: n=number of studies, ln*RR* = natural log of overall summary effect, *p*= probability that summary effect ≠ 0

^2^Funnel plot appears asymmetrical

^3^Begg and Mazumdar Kendall rank correlation: tau = rank correlation coefficient (with continuity correction), two-tailed *p* = probability that study effect sizes are correlated with their sampling variances

^4^Egger’s linear regression: *β =* intercept of regression line, *p* = probability of significant asymmetry in study effect size/study size association. The regression runs through zero if the funnel plot is symmetrical. The size of the deviation of the intercept from the origin is a measure of asymmetry, with two-tailed *p* <0.05 indicating significant asymmetry (Sterne and Egger 2005).
