# Supplementary figures and images for: Identification of Important Physiological Traits and Moderators That Are Associated with Improved Salt Tolerance in CBL and CIPK Overexpressors through a Meta-Analysis
Source: Front Plant Sci. 2017 May 29;8:856. doi: 10.3389/fpls.2017.00856 (PMC5446987; doi:10.3389/fpls.2017.00856)

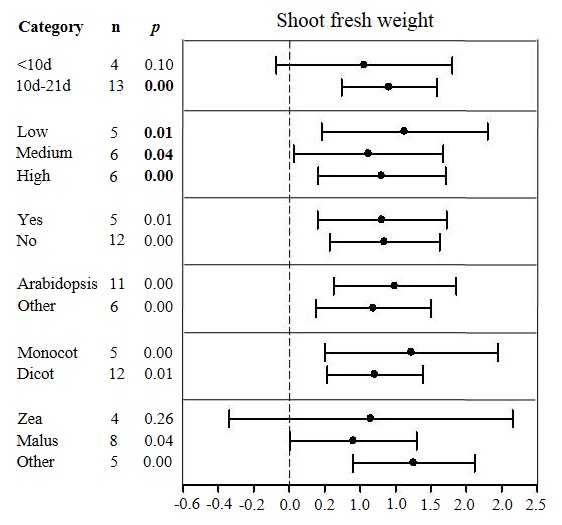

Supplement: FIGURE S1 — Weighted summary effect sizes (ln R) and 95% confidence intervals (CIs) showing how moderator variables affect the extent to which CIPK transformation modifies shoot fresh weight. A p ≤ 0.05 indicates that the moderator level was significantly different than zero; n stands for the number of studies. [file Image_1.JPEG]

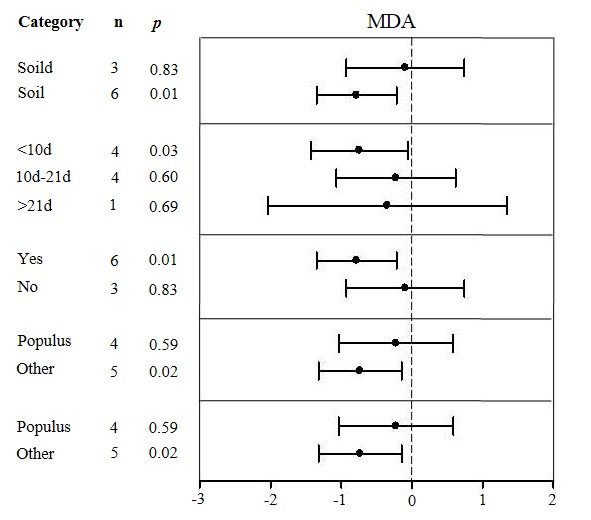

Supplement: FIGURE S2 — Weighted summary effect sizes (ln R) and 95% CIs showing how moderator variables affect the extent to which CIPK transformation modifies MDA levels. A p ≤ 0.05 indicates that the moderator level was significantly different than zero; n stands for the number of studies. [file Image_2.JPEG]

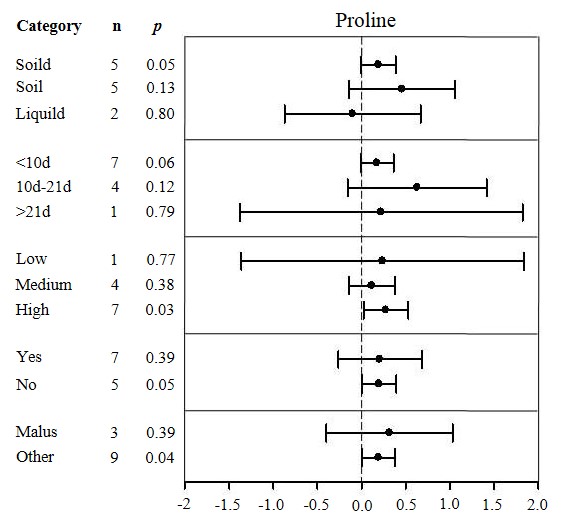

Supplement: FIGURE S3 — Weighted summary effect sizes (ln R) and 95% CIs showing how moderator variables affect the extent to which CIPK transformation modifies proline levels. A p ≤ 0.05 indicates that the moderator level was significantly different than zero; n stands for the number of studies. [file Image_3.JPEG]
